# Psychological Impacts of the New Ways of Working (NWW): A Systematic Review

**DOI:** 10.3390/ijerph17145080

**Published:** 2020-07-14

**Authors:** Yasuhiro Kotera, Katia Correa Vione

**Affiliations:** Human Sciences Research Centre, University of Derby, Derby DE22 1GB, Derbyshire, UK; k.vione@derby.ac.uk

**Keywords:** new ways of working, psychological impacts, systematic review, work engagement, work-life boundary, mental demands, fatigue, autonomy, organizational commitment

## Abstract

Digitalization of knowledge work is essential for today’s organizations, responding to diversified employee needs. Many organizations are already implementing some form of flexibility to help workers perform work and non-work duties, while maintaining high productivity. While these changes in workplaces, “New Ways of Working (NWW)”, have been discussed in the literature, a systematic appraisal of evidence of NWW has not been conducted. Relating to poor work-related mental health worldwide, this systematic review analyzed the psychological impacts of NWW, and the quality and quantity of NWW research. Following the preferred reporting items for systematic reviews and meta-analysis (PRISMA) guidelines, NWW studies targeting psychological outcomes were evaluated. Initial literature search on ProQuest, PsycINFO, Science Direct, and Google Scholar retrieved 308 titles, from which seven articles fulfilled all inclusion criteria. Our appraisal revealed that NWW research evaluated diverse psychological outcomes. While NWW can help workers’ engagement, work-related flow, and connectivity among staff, NWW can also increase blurred work-home boundary, fatigue, and mental demands. The quality of NWW research was overall medium, needing more rigorous studies. Our findings can inform decision-makers in the workplace to effectively implement NWW, and researchers to improve the quality and the usefulness of future NWW studies.

## 1. Introduction

Digitalization of knowledge work has been increasingly implemented and changed the way people work [1], supporting the United Nations’ recommendations and goals for sustainable development [2,3]. Thanks to the modern advancement of information and communication technology (ICT), the workforce can engage with work ubiquitously at any time [4]. This is particularly crucial for organizations, as the employees’ needs associated with work have been diversified due to various commitments outside work: organizations that cannot adjust their work restrictions to their employees’ work needs will have a limited chance to recruit or maintain a good workforce [5]. Accordingly, organizations and policy makers have devised and implemented more flexible and adaptive work styles to retain high productivity while actively supporting workers in non-work duties. This relatively new trend is called “New Ways of Working (NWW)” [6]. Organizations implementing NWW are characterized by a mixture of temporal flexibility (variation in the numbers of hours worked and the timing of the work; e.g., flexitime [7]), and spatial flexibility (allowing work tasks to be carried out outside the office, such as at home [8]). Therefore, NWW is commonly regarded as a work style that is flexible in time and location, supported by: (i) active use of ICT and (ii) management with clearly defined targets [9]. These two factors are essential in NWW because: (i) ICT enables workers, who work at a different time and in different spaces to collaborate, and (ii) management with clearly defined targets can compensate for a lack of face-to-face interactions.

NWW’s advantages are not limited to workforce management. NWW is thought to increase work autonomy [10], a key component of work motivation, which can then lead to increased work performance [11]. Having a sense of control over worktime can help employees manage their work-life balance [12], which is associated with good work-related mental health [13]. Digitalization of work information can help reduce costs and increase efficiency and information sharing among colleagues [14]. For example, working at home can reduce commuting time and costs, and environmental pollution [15], and can also give workers a chance to perform family duties (e.g., picking up children from school) [16].

However, disadvantages of NWW have been also noted, including loss of colleagues’ support [17], reduced autonomy caused by others’ unrealistic expectation of one’s availability [18], and compromised work-life balance due to blurred boundaries between work and private lives [14]. The impacts of NWW have been ambiguous, and to date systematic appraisal of evidence of NWW has not been conducted.

NWW is a relatively new concept; however, some dimensions of NWW have been explored independently such as flexible office design [19], telework [16], and worktime control [20]. Though these studies offered helpful findings, they missed the holistic effects of NWW that combines these dimensions [14,21,22]. Additionally, these studies did not consider how flexibility was introduced (e.g., self-choice or required by the organization, how much and what kind of flexibility an individual is given) [16]. Accordingly, this review aimed to evaluate evidence of positive and negative impacts of NWW, reviewing studies that specifically focused on NWW. Among several outcomes relevant to organizations, this review focused on psychological outcomes, corresponding to challenges many organizations face today: poor work-related mental health.

Poor mental health among workers is a cause for concern in many countries [23]. For example, in the United Kingdom, workplace mental health problems resulted in 16 million lost working days in 2016 [24], costing £65 billion to the economy annually, equivalent to 3% of the GDP [25]. Likewise, poor mental health in Dutch organizations costs €750 billion, 3% of the GDP [26]. The number of Dutch workers who take absence due to poor mental health is the highest among countries in the Organization for Economic Cooperation and Development [26]. In the European Union as a whole, the total cost of work-related depression was estimated at €620 billion yearly, consisting of productivity loss, treatment costs and disability benefit payments [27]. In the east, 60% of Japanese workers suffer from intense anxiety and stress [28], and the number of Japanese workers’ compensation claims for mental health problems increased nine times in the last two decades (200 in 2000 to 1800 in 2018 [29]). Poor work mental health is a serious problem in many countries, suggesting a need to understand the psychological aspects of workers today. Therefore, this review aimed to investigate the psychological impacts of NWW.

## 2. Materials and Methods

Modelling active knowledge translation between practitioners and researchers in medical science [30], organizational psychology research places systematic reviews as the most rigorous evaluation of evidence in the field [31]. Discussing how findings can be utilized in the workplace is essential in systematic reviews, leading to realistic workplace changes [32,33]. Therefore, the practicality and utility of our findings was explicitly discussed, in addition to the preferred method of reporting items for systematic review and meta-analysis (PRISMA [34]). The extended version of the PICO (population, intervention, control and outcomes) format [35] was used to establish the research question, defining the four key research items. In organizational research, CIMO (Context, Intervention, Mechanism, and Outcome [32]) is another common format; however, because our target outcomes are psychological, we decided to use the extended PICO. The main research questions were: (i) what are the positive and negative psychological impacts of the New Ways of Working? and (ii) what quantity and quality of evidence has been reported?

### 2.1. Literature Search

Based on the guidance given by a subject librarian, the following electronic research databases were used for our comprehensive literature search [36]: ProQuest, PsycINFO, Science Direct, and Google Scholar via EBSCO. We focused on where, when, who, how, what, and why during our searches [37]. Articles published before 31 March 2020 were considered for this review (searched in April 2020). Three-hundred and nine articles were retrieved with search terms “new way? of working” and “NWW”. The title and abstract of these articles were reviewed, and 18 articles were selected for further examination. The first author completed the literature search, which was then reviewed by the second author and an external reviewer who was an organizational psychologist. Additionally, manual reference searches of a previous systematic review on remote work [38] were conducted, because there was no systematic review of NWW; no additional article was found.

### 2.2. Selection of Studies and Outcomes

Inclusion criteria for articles to be analyzed further were: (i) being published in a peer-reviewed scholarly journal written in English language, (ii) reporting an empirical study (e.g., cross-sectional study, intervention study) and/or qualitative research study (using an appropriately implemented qualitative analytical technique) of NWW, and (iii) including full-time or part-time workers who work more than three days a week as participants. Exclusion criteria were: (i) not empirical (e.g., discussion paper or research protocols), (ii) employing an N-of-1 design (i.e., case studies), and (iii) not assessing psychology outcomes (Table 1).

### 2.3. Data Extraction and Synthesis

The first and second authors reviewed all the search results; if the title of the article indicated suitability regarding the eligibility criteria, the articles were shortlisted for possible inclusion (*n* = 18). An external reviewer who was an organizational psychologist reviewed the selection process to examine if there was any potential bias. Once the external reviewer had reviewed the selection process, full texts of the shortlisted articles were examined by both co-authors independently, who then discussed, to confirm which studies met the eligibility criteria. Forward and backward reference searches of relevant articles did not find any additional eligible studies.

Key research information of included studies was extracted using a format designed by Sturt et al., 2012 [40]: publication details (authors, year, and country), study design and setting, participant characteristics, details of demographic data, intervention details, intervention facilitator, outcome measures, and study findings (see Table 2).

### 2.4. Quality Scoring: Assessing the Risk of Bias

The quality of non-randomized intervention studies was assessed using the Newcastle-Ottawa Scale (NOS), because the NOS was designed to assess the risk of bias in non-randomized intervention studies [41]. NOS considers: (i) representativeness of study group selection (four stars maximum), (ii) comparability of groups (two stars maximum), and (iii) ascertainment of either the finding or outcome of interest (three stars maximum): each study was assessed from 0 to 9 stars (the higher score indicates the lower risk: high risk was indicated by a score 0–3, medium risk was indicated by a score 4–6, and low risk was indicated by a score 7–9). Because NOS was originally introduced to assess medical research, some parts were adjusted to be used in this psychological review [42]. First, the word ‘exposure’ was changed to ‘intervention’ (e.g., ‘Ascertainment of intervention’). Second, the fourth scale item (in the ‘Selection’ category) was changed from ‘Demonstration that outcome of interest was not present at start of study’ to ‘Demonstration that the measured outcome was assessed before the intervention’, because some psychological outcomes would be present prior to the intervention (e.g., stress). Lastly, instead of medical records, a point was given if the outcome was measured using a validated psychometric scale in the first ‘Outcome’ item (‘Assessment of Outcome’).

The quality of cross-sectional studies was assessed using the adjusted version of NOS [43]. Similar to the original NOS, this version of NOS consists of three sections—selection (five stars maximum), comparability (two stars maximum), and outcome (three stars maximum)—ranging from 0 to 10 stars in total (high risk was indicated by a score 0–3, medium risk was indicated by a score 4–6, and low risk was indicated by a score 7–10).

Lastly, the quality of qualitative studies was assessed using the Critical Appraisal Skills Program (CASP) checklist [44], comprising ten items regarding research design and reporting (high risk was indicated by a score 0–4, medium risk was indicated by a score 5–8, and low risk was indicated by a score 9–12).

Both co-authors evaluated the quality of each study independently, and any disagreement was discussed in order to come to an agreement. 

## 3. Results

### 3.1. Search Results

**Figure 1:**
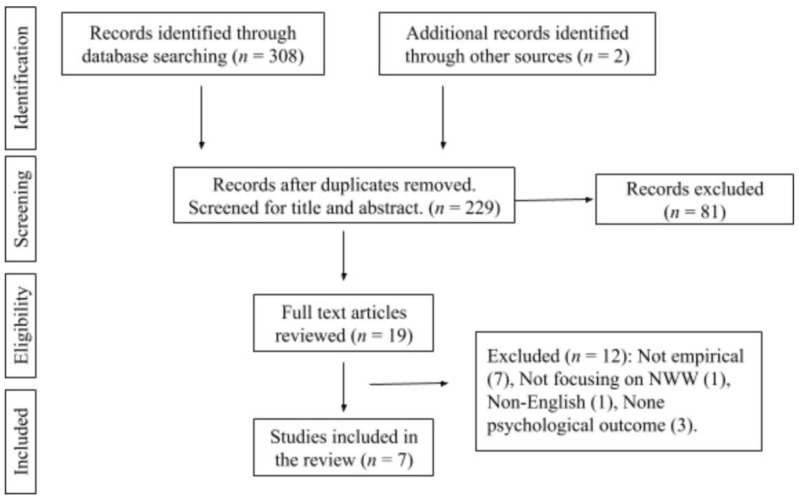
PRISMA flow diagram of the article selection process.

The initial comprehensive literature search retrieved 308 articles in total. Expert consultation with two senior lecturers in organizational psychology and business management identified two additional articles. After removing 81 duplicate results, titles and abstracts of 229 articles were reviewed, which then identified 20 articles for a full-text review. Finally, a total of seven articles met all the eligibility criteria (See Table 2 for included articles and Table 3 for excluded studies). Figure 1 shows the PRISMA flow diagram for the article selection process.

### 3.2. Characteristics of Included Studies

Of the seven included studies, six were quantitative studies [9,10,22,45,46,47] and one was a qualitative study [45]. In the quantitative studies, three studies were cross-sectional [22,46,48], two were pre-post within-subject studies [10,47], and one was a quasi-experimental study [9]. The qualitative study employed semantic content analysis [45]. Six studies were conducted in the Netherlands [9,10,22,46,47,48] and one in Slovakia [45].

Because the psychological outcomes explored were diverse, we classified these psychological outcomes into cognitive, emotional and social outcomes, using the adapted version of Keyes’ categorization of mental health [61] (Table 4). Included studies examined these three subcategories of psychological outcomes in a balanced manner: four studies explored cognitive outcomes [9,10,45,47], six studies explored emotional outcomes [9,10,22,46,47,48] and social outcomes [9,22,45,46,47,48], respectively. Among the cognitive outcomes, job demands were examined in two studies [9,10]; among the emotional outcomes, work engagement was examined in three studies [10,46,48], and employee satisfaction was examined in two studies [9,47]; six studies that explored social outcomes focused on communications/interactions/contacts [8,9,22,46,47] and family support [45]. Lastly, the level of NWW was measured in three studies using a five-item scale developed in collaboration with the organization (α = 0.56−0.84) [10], a ten-item scale covering the five NWW facets (α = 0.86) [46], and the total hours spent on: (i) remote access, (ii) working at home, (iii) email, and (iv) phone, which were identified after an HR-manager interview and reviewing the company’s NWW policy (α = 0.70) [48] (Table 5).

### 3.3. Risk of Bias

The risk of bias for the three nonrandomized controlled studies (two pre-post within-subject studies [10,45] and one quasi-experimental study [9]) was deemed to be high to low (Table 6). Nijp et al.’s quasi-experimental study [9] scored 8 out of 9, indicating low risk of bias, while Blok et al.’s pre-post within-subject study [47] scored 0 out of 9, showing high risk of bias. All three studies did not report on the representativeness of their exposed samples.

The risk of bias for the three cross-sectional studies [22,46,48] was deemed high to medium (Table 7). All three studies used both psychometrically validated scales and non-validated scales that described how they were made. All three studies also assessed outcomes using self-report measures. While the number of non-responders was reported, none of the studies referred to the comparability between responder and non-responder characteristics.

Lastly, the risk of bias for the qualitative study [45] was deemed medium (7 out of 12; Table 8). Though the research aims, methods, design, recruitment, data collection, and findings were clearly reported, the researcher-participant relationship, ethical consideration and the details of the data analysis were not reported.

## 4. Discussion

This systematic review adhered to the PRISMA guidelines and appraised the quality of evidence regarding studies focused on and evaluating NWW. A total of seven studies (six quantitative and one qualitative), recruiting 2431 worker participants altogether, fulfilled all of the eligibility criteria for in-depth review and assessment. Findings illustrate that NWW research evaluated diverse psychological outcomes in a balanced manner: cognitive, emotional, and social outcomes. While NWW can help workers’ work engagement, work-related flow, and connectivity among staff, NWW can also impact workers’ psychological aspects negatively including blurred work-home boundary, fatigue, and mental demands. While these findings can inform organizations worldwide today (especially because many workers have to socially isolate themselves due to the COVID-19 pandemic), the findings need to be interpreted with caution because the quantity of evidence was not high, and the quality of evidence varied from low to high.

This is the first systematic review to assess the psychological impacts of NWW, which can inform many organizations worldwide today that attempt to address the poor mental health of their workers. The seven included studies were all conducted in Europe, six being in the Netherlands. Considering the term NWW was developed in the Netherlands, it is understandable that many Dutch organizational scientists have explored it; however, this part of the findings suggests that NWW needs to be evaluated in other countries as well. Especially, countries that are culturally different from European countries and have many mentally distressed workers are needed to make a fair comparison. For example, Japan may be suitable for this comparison, as this country has been more actively working towards changing its working culture (e.g., Work-Style Reform [62]). For example, following this national trend, Microsoft Japan implemented a four-day week, using ICT, and successfully increased their productivity by 40% [63]. How NWW can be used (i.e., what types of adjustments may be needed) in organizations in other countries needs to be examined.

Whilst the positive psychological impacts of NWW—e.g., higher work engagement, work-related flow, and connectivity among staff—can attract many organizations to consider implementation of NWW, the negative impacts such as blurred work-home boundary, fatigue, and mental demands should be addressed when/if NWW is implemented. Family’s and company’s understanding of working style are essential for the success of NWW [45], therefore an introductory meeting to explain working conditions is recommended both at work and at home [64]. Despite companies’ ergonomic guidelines for working from home reported in Nijp et al.’s study [9], increased fatigue and mental demands may suggest a need for wellbeing measures. For example, online morning huddles (a brief socialization gathering among staff to check on how everyone is at the start of a workday) were effective for increasing wellbeing of university lecturers working remotely [65]. Furthermore, the season may change these effects (fall > spring) [66]), e.g., a short walk was helpful for mental health, reducing fatigue in German workers from various sectors [67]. When an organization implements NWW, it also needs to support its employees’ wellbeing in working at home. Workers should be informed of these helpful wellbeing measures before implementing NWW.

While the included studies examined diverse psychological outcomes, there was no randomized controlled trial and only one quasi-experimental study; rigorous research design was not commonly used in NWW research. Indeed, in the organizational setting randomized controlled trials may not be practical and helpful for participating workers [42]. However, more experimental studies should be performed to demonstrate the effects of NWW through rigorous examination. Likewise, the risk of bias in the included studies was medium overall. Only one study was assessed as low risk of bias [9], four studies as medium risk of bias [10,45,46,48], and two as high risk of bias [22,47]. In line with a previous observation [20], NWW needs to be appraised in more rigorously designed research. The one study that was assessed as low risk of bias [9] only explored employees in a Dutch financial company and did not explore key NWW-related constructs such as work engagement, stress and work-life balance [14]. Future research needs to explore more diverse workforces regarding these key NWW constructs. As noted above considering the current COVID-19 pandemic situation, not only diverse workforces but also the contexts of implementing NWW should be discussed in future NWW research (i.e., whether it is required by the country or organization; whether it is arbitrary and an employee can choose to utilize NWW; how the organization or manager introduced and justified NWW to staff). Moreover, there is no validated psychometric scale to measure NWW: the development of an NWW scale needs to take place.

Though this review offers helpful insights into the psychological impacts of NWW, several limitations should be noted. First, unpublished findings or studies published in other languages than English were excluded; there may be additional evidence here of the psychological impacts of NWW. Second, all quantitative measures were self-reported scales, therefore social desirability bias might have been present [68]. Lastly, the number of included studies was only seven, making it difficult to arrive at a firm conclusion. Although our search included three databases and Google Scholar, it is possible that relevant publications may have been missed if these were only indexed in other databases.

## 5. Conclusions

The seven selected articles, all conducted in Europe, in this systematic review demonstrate that NWW research evaluated a wide range of psychological outcomes, and NWW can impact on positive psychological outcomes (e.g., work engagement, work-related flow, and connectivity among staff) and negative ones (e.g., blurred work-home boundary, fatigue, and mental demands). Organizations need to address these negative psychological impacts of NWW, supporting employees’ wellbeing when/if implementing NWW within their organizations. Overall the quality of NWW research was medium, needing more methodologically rigorous research. Likewise, as noted as a limitation, the quantity of NWW research can also be improved: there were only seven studies included, limiting the reliability of the findings. This review identified that future NWW research should: (i) recruit diverse populations, (ii) evaluate relevant psychological outcomes, and (iii) develop a validated scale to measure NWW. It is important to note that COVID-19 may have introduced NWW in different sectors without adequate planning and execution, therefore future studies also need to address how to mitigate negative impacts of NWW after its implementation if this is to become part of the post-pandemic workplace reality. Our findings can help decision-makers at a workplace to effectively implement NWW, and researchers to improve the quality and the usefulness of future NWW studies.

## Figures and Tables

**Table 1:** Extended PICO (population, intervention, control and outcomes) for this review.

Research Question	What Are the Advantages and Disadvantages of NWW? What Quantity and Quality of Evidence Has Been Reported?	
	Inclusion Criteria	Exclusion Criteria
Population	Workers in an organization (i.e., employees aged 18 years or older)	Workers who are less than 18 years old and non-work samples
Intervention	New Ways of Working (NWW) *	Other work arrangements than NWW
Comparator	Any comparator including no intervention	
Outcomes	Psychological outcomes	Other outcomes
Study Design	Empirical, quantitative and/or qualitative study	N-of-1 studies, reviews, discussion articles, articles introducing theories/concepts/models/applications
Others	Published in a peer-reviewed academic journal in English, published before 31 March 2020.	

* New Ways of Working (NWW) = Work arrangements, where workers have autonomy over their work time and location, while being supported by media technologies [14]. Articles need to specifically focus on NWW: studies that explored part of NWW were excluded (e.g., [39]).

**Table 2:** Study details of selected articles exploring New Ways of Working.

No.	Author(s), Year. Country.	Sample and Setting	Study Design	Measures or Outcomes	Findings
1	Van Steenbergen et al., 2018. Netherlands [10].	126 employees (82 women & 44 men; Age 39.5 ± 8.7) of a large financial services provider.	Intervention pre-post study with 3 assessment points (1 assessment before NWW, and 2 assessments after NWW 3 and 12 months later). Impacts on job demands, job resources, burnout and work engagement, and how Psychological Capital (PsyCap) affects these changes.	New ways of working, Job demands, Job resources, Maslach Burnout Inventory, Utrecht Work Engagement Scale (UWES), Psychological Capital questionnaire	NWW decreased mental demands, workload, autonomy, and possibilities for professional development, while did not harm relationships with supervisors. Burnout and work engagement remained stable. The effects did not depend on PsyCap.
2	Fedakova & Istonova, 2017. Slovakia [45].	23 IT workers (14 men & 9 women; Age 33.64 ± 3.03 for men; 33.44 ± 3.57 for women).	9 focus group-structured interviews with semantic content analysis	Work-family boundary, organizational support, family support	NWW blurred psychological borders between work and family and intensified boundary-spanning thoughts. Tele-homework had more advantages than disadvantages. Organizational and family support is critical for success of NWW.
3	Gerards et al., 2017. Netherlands [46].	656 employees a wide range of sectors, excluding managers. Gender and age were not reported.	Cross-sectional study explored relationships among NWW, work engagement, social interaction and transformational leadership	NWW, Utrecht Work Engagement Scale (UWES), social interaction and transformational leadership	Three facets of NWW—management of output, access to organizational knowledge, and a freely accessible open workplace—positively affected work engagement. The latter two facets were mediated by social interaction and transformational leadership.
4	Nijp et al., 2016. Netherlands [9].	441 workers (269 men & 172 women; Age 43.85 ± 9.42) at a financial company divided into NWW group (*n* = 361) and non-NWW group (*n* = 80)	Quasi-experimental design with 3 assessment points (one month before, 4 months and 10 months after implementation of NWW).	Worktime control, work hours, job autonomy, job demands, social contact with colleagues, social contact with supervisors, Survey Work-Home Interaction Nijmegen, Fatigue Assessment Scale, and job-related outcomes (performance, organizational commitment and job satisfaction)	While the levels of fatigue and health reduced in NWW group, these increased in non-NWW group. Significant interaction effects in health.
5	Peters et al., 2014. Netherlands [22].	1017 employees and their line managers (*n* = 89), across 89 job categories in 30 organizations.	Cross-sectional study explored relationships among work-related flow, telework, empowerment, collegial support and leadership	Work-Related Flow Inventory, implemented employee empowerment, perceived employee empowerment, telework frequency, supporting leadership, collegial support, collegial commitment	Anticipated effects on work-related flow (particularly work enjoyment) are not achieved when employees themselves do not experience being empowered, and when they do not use and experience their working conditions as job resources (home-based teleworking and trust relationships characterized by supporting leadership, collegial support, and collegial commitment).
6	Blok et al., 2012. Netherlands [47].	58 employees (34 men and 24 women; Age M = 45 years) responded to the first questionnaire (baseline), and 52 employees (28 men and 24 women; Age M = 44 years) responded to the second questionnaire (6 months after NWW). A total of 39 participants filled out both questionnaires.	Intervention pre-post study (baseline and 6 months after implementing NWW).	Work behavior (i.e., work location, work times and a change towards NWW management style) and the effect on business objectives such as knowledge sharing, employees satisfaction, and collaboration.	NWW role model and focus on results improved (mean comparison).
7	ten Brummelhuis et al., 2012. Netherlands [48].	110 telecom workers (62 men & 48 women; Age 42.5 ± 8.9 years)	5-day diary study exploring the effects of NWW on work engagement and exhaustion, and whether communication quality mediated these relationships.	NWW, UWES, Utrecht Burnout Scale (exhaustion), communication quality, connectivity among coworkers.	NWW was positively related to work engagement due to increased effective and efficient communication. NWW was also positively associated with communication quality, and connectivity among coworkers, but not associated with work exhaustion.

**Table 3:** Reasons for exclusion.

Article	Reason
Jemine et al., 2020 [49]	Not psychological outcomes
Mache et al., 2020 [50]	Not focusing on NWW
Jemine et al., 2019 [51]	Not psychological outcomes
Kingma, 2019 [52]	Not empirical
Procter et al., 2016 [53]	Not psychological outcomes
Coun & Gelderman, 2015 [54]	Non-English (only title and abstract were in English)
Hollingsworth, 2009 [55]	Not empirical
Morris et al., 2009 [56]	Not empirical
Osborn & Smyth, 2009 [57]	Not empirical
Vize, 2009 [58]	Not empirical
Morris & Nixon, 2008 [59]	Not empirical
Baguley et al., 2007 [60]	Not empirical

**Table 4:** Outcomes explored in the included studies.

	Study	Cognitive	Emotional	Social
1	Van Steenbergen et al., 2018 [10]	Mental demands (+); Autonomy (+)	Burnout; Work engagement; Psychological capital	
2	Fedakova & Istonova, 2017 [45]	Work-family boundary (−)		Organizational support (+); Family support (+)
3	Gerards et al., 2017 [46]		Work engagement (+)	Social interaction (+); Transformational leadership (+)
4	Nijp et al., 2016 [9]	Worktime control; Job demands (+); Work-home interaction; Fatigue (+)	Organizational commitment (+); Job satisfaction	Social contact with colleagues and supervisors
5	Peters et al., 2014 [22]		Flow (+); Work empowerment (+)	Supporting leadership (−); Collegial support (−); Collegial commitment (−)
6	Blok et al., 2012 [47]	Knowledge sharing (−)	Employee satisfaction	Collaboration
7	ten Brummelhuis et al., 2012 [48]		Work engagement (+); Exhaustion	Communication quality (+); Connectivity among coworkers (+)

(+) positive relation with NWW; (−) negative relation with NWW.

**Table 5:** Scales to measure the New Ways of Working.

**New Ways of Working (α = 0.56–0.84; Van Steenbergen et al., 2018 [10]). Response: 1 = Totally disagree to 7 = Totally agree**	
**1. I decide for myself where (office, home, elsewhere) and when I work.** **2. I use information technology (e.g., smartphone, laptop), so I can work at any chosen location or time.** **3. I regularly work remotely with my colleagues and partners.** **4. In our office, I work in an ‘activity-related’ manner (e.g., using spaces for concentration, communication, meetings).** **5. I do not have my own personal desk (flex-desk concept).**	
**New ways of working (α = 0.86; Gerards et al., 2017 [46]). Response: 1 = Never to 7 = Always**	
**Items**	**Corresponding NWW facet**
1. I am able to set my own working hours. 2. I am able to determine where I work.	Facet 1: Time- and location-independent work
3. I am able to determine the way I work.	Facet 2: Management of output
4. I can access all necessary information on my computer, smartphone, and/or tablet. 5. I am able to reach colleagues within the team quickly. 6. I am able to reach managers quickly. 7. I am able to reach colleagues outside the team quickly.	Facet 3: Access to organizational knowledge
8. I have the ability to adapt my working scheme to my phase of life and ambitions.	Facet 4: Flexibility in working relations
9. The building is arranged so that colleagues are easily accessible. 10. The building is arranged so that managers are easily accessible.	Facet 5: Freely accessible open workplace
**New ways of working (α = 0.70; ten Brummelhuis et al., 2012 [48]).**	
NWW was assessed as the hours using: (i) remote access, (ii) working at home, (iii) email, and (iv) phone, based on the HR-manager interview and the company’s NWW policy. Specific items were not reported.	

**Table 6:** Assessment of risk of bias for intervention studies.

Bias Category	Selection				Comparability	Outcome			
Author, Year	Representativeness of Exposed Cohort	Selection of Non-Exposed Cohort	Ascertainment of Intervention	Demonstrate Outcome Assessed Before Intervention	Comparability of Cohorts on Basis of Design (*) or Analysis (*)	Assessment of Outcome	Follow-Up Long Enough	Adequacy of Follow-Up	Number of Stars (0–9)
Quasi-experimental study									
Nijp et al., 2016 [9].		*	*	*	**	*	*	*	8
Within-subject pre-post study									
Van Steenbergen et al., 2018 [10].		NA	*	*	NA	*	*	*	5
Blok et al., 2012 [47].		NA			NA				0

* Quality scoring: representativeness of study group selection (four stars maximum), comparability of groups (two stars maximum), and ascertainment of either the finding or outcome of interest (three stars maximum); maximum of 9 stars for each study, the higher score indicates the lower risk: high risk was indicated by a score 0–3, medium risk was indicated by a score 4–6, and low risk was indicated by a score 7–9.

**Table 7:** Assessment of risk of bias for cross-sectional studies.

Bias Category	Selection				Comparability	Outcome		
Author, Year	Representativeness of Sample	Sample Size	Non-Respondents	Ascertainment of the Exposure (Risk Factor) **	The Subjects in Different Outcome Groups are Comparable, Based on the Study Design or Analysis (*). Confounding Factors Are Controlled (*).	Assessment of Outcome **	Statistical Test	Number of Stars (0–10)
Gerards et al., 2017 [46].	*			*	*	*	*	5
Peters et al., 2014 [22].				*		*		2
ten Brummelhuis et al., 2012 [48].		*		*	*	*	*	5

* Quality scoring: representativeness of study group selection (four stars maximum), comparability of groups (two stars maximum), and ascertainment of either the finding or outcome of interest (three stars maximum); maximum of 9 stars for each study, the higher score indicates the lower risk: high risk was indicated by a score 0–3, medium risk was indicated by a score 4–6, and low risk was indicated by a score 7–9.

**Table 8:** Assessment of risk of bias for qualitative research.

Quantitative Studies	Clear Statement of Aims	Appropriate Methodology	Appropriate Research Design	Appropriate Recruitment	Data Collection Addressed Research Issues	Researcher-Participant Relationship Considered	Ethical Issues Considered	Rigorous Data Analysis	Clear Statement of Findings	How Valuable Is the Research? (0–3)	Score (0–12)
Fedakova & Istonova, 2017 [45].	Y	Y	Y	Y	Y	N	N	N	Y	1	7

Y = Yes, N = No.
